# Expression of RCAS1 Correlates with Urothelial Bladder Cancer Malignancy

**DOI:** 10.3390/ijms16023783

**Published:** 2015-02-10

**Authors:** Wojciech Jóźwicki, Anna A. Brożyna, Jerzy Siekiera, Andrzej T. Slominski

**Affiliations:** 1 Department of Tumor Pathology and Pathomorphology, the Ludwik Rydygier Collegium Medicum in Bydgoszcz, Nicolaus Copernicus University in Toruń, Romanowska Street 2, Bydgoszcz 85-796, Poland; E-Mail: anna.brozyna@cm.umk.pl; 2 Department of Tumor Pathology and Pathomorphology, the Franciszek Łukaszczyk Oncology Centre, Romanowska Street 2, Bydgoszcz 85-796, Poland; 3 Department of Urology, the Franciszek Łukaszczyk Oncology Centre, Romanowska Street 2, Bydgoszcz 85-796, Poland; E-Mail: siekieraj@co.bydgoszcz.pl; 4 Department of Pathology and Laboratory Medicine, University of Tennessee HSC, 930 Madison Avenue, Memphis, TN 38163, USA; E-Mail: aslominski@uthsc.edu; 5 Department of Dermatology, University of Alabama at Birmingham, Birmingham, AL 35294, USA

**Keywords:** RCAS1, urothelial bladder cancer, tumor microenvironment, immune escape

## Abstract

RCAS1 is a protein that participates in regulation of the tumor microenvironment and its immune responses, all in order to evade the immune system. The aim of this study was to analyze RCAS1 expression in urothelial bladder cancer cells (and in fibroblasts and macrophages of the tumor stroma) and its relationship with the histological pattern of malignancy. Eighty-three postcystectomy patients were enrolled. We analyzed the histological maturity (grade), progress (pT stage), tissue invasion type (TIT), nonclassic differentiation number (NDN), and the ability to metastasize (pN). The expression of RCAS1 protein was analyzed by immunohistochemistry. Indicators of histological malignancy were observed solely in association with the RCAS1 expression in cells in the border parts (BPs) of the tumor. Histological malignancy of the tumor, indicated by the pT and pN, and metastasis-free survival time, correlated significantly with RCAS1 expression in tumor neoplastic cells, whereas malignancy determined by grade, TIT, and NDN correlated with RCAS1 expression in fibroblasts and macrophages in the tumor microenvironment. These findings suggest that the increased RCAS1 expression depends on its cellular source and that RCAS1 expression itself is a component of various signaling pathways. The immune escape occurs within the tumor BPs, where the increase in the RCAS1 expression occurs within tumor cells and stromal cells in its microenvironment. We conclude that the histological pattern of tumor malignancy, indicated by grade, TIT, NDN, pT, and pN is a morphological indicator of immune escape.

## 1. Introduction

A tumor malignancy and its relation to histological structures is a part of histopathological diagnosis. This relationship is associated with histological immaturity and disorganization of the tumor, which is defined by the differences between neoplastic histological structures and normal tissue [1,2]. The histological maturity of a tumor usually indicates lower potential for malignancy. The classification of tumor malignancy uses the criterion of histological maturity [1,2], in which a higher malignancy, indicated by G, is assigned to less mature (more anaplastic) tumors. However, histological grading is not a sufficient indicator of the tumor behavior.

Among urothelial tumors of the urinary bladder, a group with nonclassic, variant differentiation can be distinguished as tumors in which the malignancy grade correlates with the prominence of a nonclassic structure of the tumor [3,4,5,6,7,8,9,10,11,12]. The most common histological variant is squamous, followed by glandular. Further histological variants include nested, microcystic, micropapillary, sarcomatoid and clear cell variants, and others. Urothelial bladder cancers showing divergent differentiation are also accompanied by a high grade of malignancy and high stage of advancement of the tumor [12].

Several studies, including our earlier reports, found that nonclassic differentiation in bladder cancers is related to a more aggressive disease course, even if only traces of divergent histological structures are present [13,14,15]. We also reported previously that the number of nonclassic types of differentiation, as indicated by the nonclassic differentiation number (NDN), reflected the capacity for multidirectional differentiation, even in vestigial extensions, and was an important new criterion for estimating tumor malignancy [14,15] and the ability of a tumor to metastasize [16]. The World Health Organization (WHO) and International Agency for Research on Cancer (IARC) have accepted that the pattern of tumor growth has prognostic significance and that frontal growth has a more favorable prognosis than tentacular growth [12].

In our previous studies of urothelial bladder cancers, we identified frontal, focal, styloid, and dispersive models of tumor growth (defined by the tissue invasion type (TIT)), and we found that TIT characterized tumor malignancy and reflected the degree of local aggressiveness [17,18]. In our earlier studies [19], we observed differential expression of receptor-binding cancer antigen expressed on SiSo cells (RCAS1) in the border parts (BPs) and central parts (CPs) of ovarian tumors. We defined BPs as younger parts of the tumor that showed signs of dynamic growth and CPs as older parts that did not show signs of dynamic growth [19]. RCAS1 is involved in generating the suppressive profile of the tumor microenvironment, which allows cancer cells to evade immune surveillance. We found that the level of RCAS1 in cancer cells and the number of RCAS1-positive macrophages was higher in the BPs of tumors. Higher expression of RCAS1 was related to a lack of response to chemotherapy [19]. Similarly, other authors have observed greater expression of metallothionein, which has proproliferative and antiapoptotic activities, within the BPs of pharyngeal squamous cell carcinomas [20]. The commonly accepted indicators of urothelial cancer histological malignancy also include the pathological stage (pT) and presence of metastases (pN > 0). These findings raised the question as to what factors determine the histological pattern of the tumor malignancy.

New concepts to explain the promotion of neoplastic growth consider the advantages of tumor development of a molecular “dialogue” between a cancer cell and its microenvironment [21,22,23], which eventually generates a space for safe tumor growth. The idea of immune escape [24] is supported by recent studies suggesting that some elements of the immune system may have a protective influence on neoplastic growth [25,26]. The interaction between a tumor and the immune system requires several molecular regulators. One of the most important one appears to be RCAS1 [27,28], of which cell expression correlates with a poor prognosis according to some authors [29,30]. RCAS1 is a protein that is encoded in humans by the *EBAG9* gene and participates in the immunomodulatory effects of tumor cells. This may occur through the induction of cell cycle arrest and/or apoptosis in RCAS1 receptor-bearing human cells [24,31], which may lead to immune escape.

The aim of this study was to analyze RCAS1 expression in neoplastic cells of urothelial bladder cancer and in tumor-associated macrophages (TAMs) and cancer-associated fibroblasts (CAFs) of the tumor’s microenvironment. We assessed the relationship between RCAS1 expression and components of the histological pattern of tumor malignancy including the tumor grade, pT, TIT, NDN, and the ability to metastasize.

## 2. Results

### 2.1. Characteristics of RCAS1 Expression within Cancer Cells

**Figure 1:**
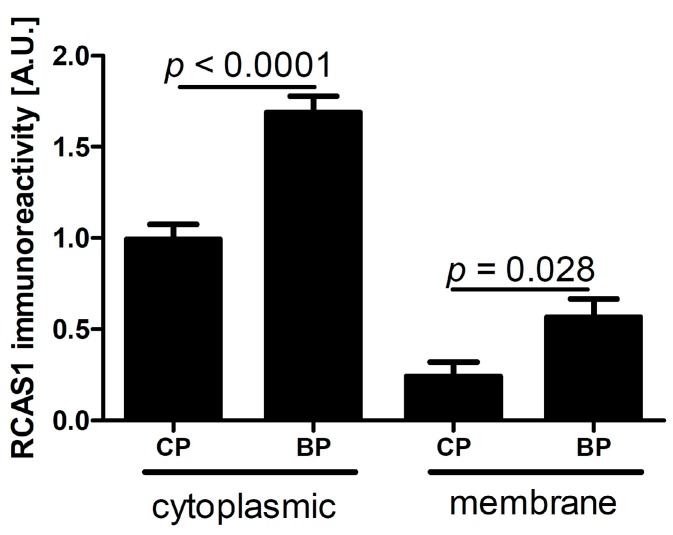
RCAS1 immunoreactivity in tumor cells in the central part (CP) and border part (BP) regions of tumors. Significant differences are denoted by *p* values (*t* test).

**Figure 2:**
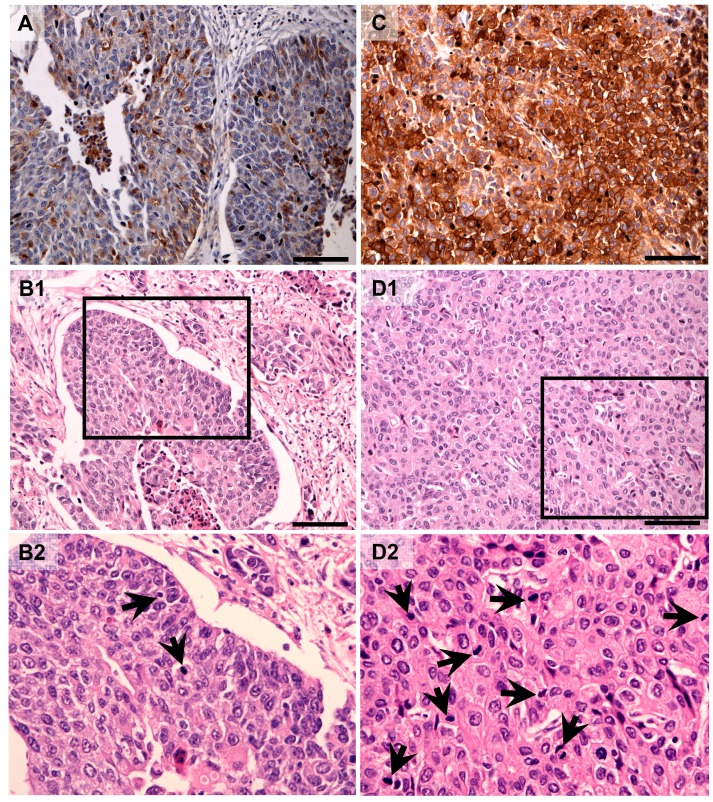
Representative RCAS1 immunolocalization in central parts (**A**) (low mitotic index, **B-1**, **B-2**) and border parts (**C**) (high mitotic index, **D-1**, **D-2**) of the tumors. Figure 2B-2 and Figure 2D-2 present the fragments indicated by the black squares within Figure 2B-1 and Figure 2D-1, respectively. Arrows indicate mitosis. Scale bars: 100 µm.

Cytoplasmic or membrane immunoexpression of RCAS1 was detected. The intensity varied by location and was related to the tumor stage and an ability to metastasize. In general, cancer cells showed intense cytoplasmic and membrane immunoreactivity within the tumor border parts (BPs) (Figure 1 and Figure 2). Similarly, RCAS1 presence was related to proliferation activity and both for CPs and BPs cytoplasmic RCAS1 level was positively correlated with mitotic index (Spearman Rank = 0.32, *p* ≤ 0.05, and Spearman Rank = 0.30, *p* ≤ 0.05, respectively).

Table 1 summarizes relationship between RCAS1 and pathological markers of tumor behavior. Detailed discussion is provided below.

**Table 1:** The presence (+) or absence (–) of statistical associations between tissue RCAS1 expression and pathological characteristics of urothelial bladder cancer. BP: border part of tumor; CP: central part of tumor; TAMs: tumor-associated macrophages; CAFs: cancer-associated fibroblasts. TIT-tissue invasion type, NDN-nonclassic differentiation number, pT-pathological assessment of primary tumor extent, pN-pathological assessment of metastases to nearbyregional lymph nodes.

Type of Expression and Number of Cells	Localization within Tumor	Grade	TIT	NDN	pT	pN
Membrane expression	BP cancer cells	−	−	−	+	+
					*p* = 0.008	*p* = 0.027
	CP cancer cells	−	−	−	−	−
Cytoplasmic expression	BP cancer cells	−	−	−	−	+
						*p* = 0.019
	CP cancer cells	−	−	−	−	−
	BP TAMs	−	+	+	−	−
			*p* = 0.039	*p* = 0.010		
	CP TAMs	–	–	–	−	−
	BP CAFs	+	+	+	−	−
		*p* = 0.040	*p* = 0.004	*p* = 0.016		
	CP CAFs	−	−	–	−	−
Number of cells	BP TAMs	+	−	+	–	−
		*p* = 0.032		*p* = 0.008		
	CP TAMs	−	−	–	−	−
	BP CAFs	−	−	–	+	−
					*p* = 0.035	
	CP CAFs	−	−	–	–	−

#### 2.1.1. RCAS1 Immunoreactivity Relative to Tumor Stage

**Figure 3:**
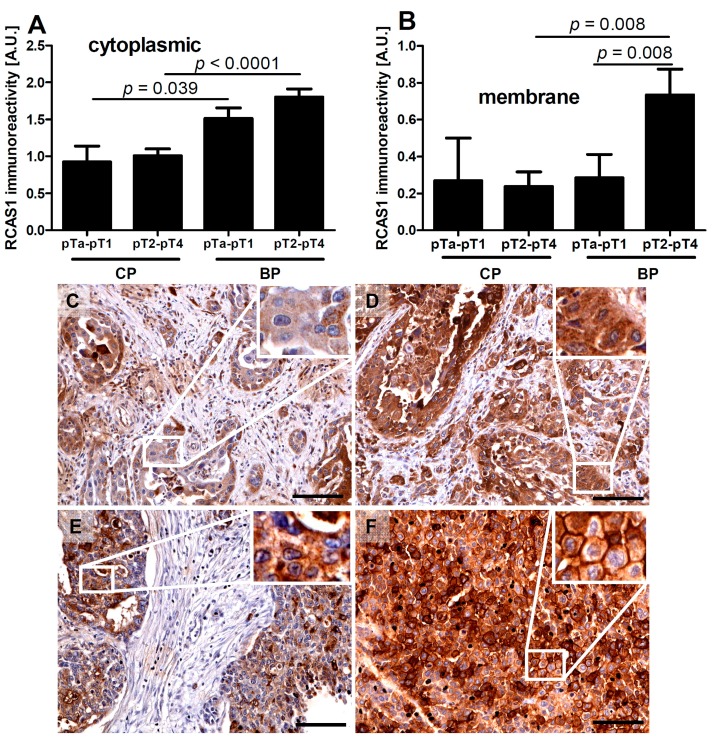
Relationships between RCAS1 cytoplasmic (**A**) and membrane (**B**) immunoreactivity of non-muscle-invasive bladder cancer (stages pTa, pTis, and pT1) and muscle-invasive bladder cancer (stages pT2–pT4). Representative immunostaining of cytoplasmic RCAS1 in the central part (CP) (**C**) and border part (BP) (**D**) of pTa–pT1 and cytoplasmic and membrane RCAS1 in the CP (**E**) and BP (**F**) of pT2–pT4. White squares indicate the areas enlarged in the insets. Scale bars: 100 µm.

Significant differences in cytoplasmic RCAS1 expression were found between cells of the central part (CP) and BP regions of both non-muscle-invasive bladder cancer (pTa–pT1) and muscle-invasive bladder cancer (pT2–pT4) (Figure 3A). The membrane expression of RCAS1 was also significantly higher within the BP region compared with the CP region of pT2–p4 (Figure 3B). The membrane and cytoplasmic RCAS1 immunostaining in the BP and CP of MICB and pTa-pT1 is shown in Figure 3C–F.

#### 2.1.2. RCAS1 Immunoreactivity Relative to Metastasizing Capacity

**Figure 4:**
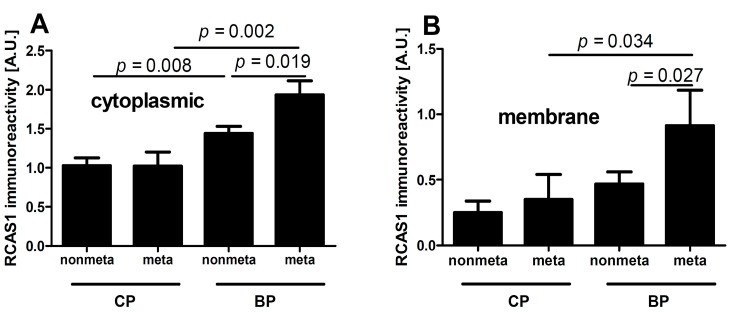
Relationship between cytoplasmic (**A**) and membrane (**B**) RCAS1 immunoreactivity of nonmetastasizing (nonmeta) and metastasizing (meta) bladder cancers.

The cytoplasmic and membrane expression of RCAS1 was significantly higher within the BPs of metastasizing tumors (Figure 4) compared to the CPs.

**Figure 5:**
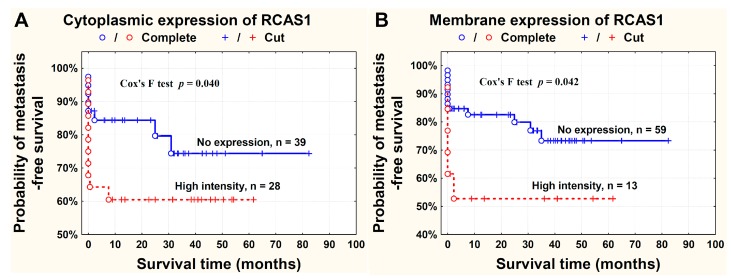
Probability of metastasis-free survival time in relation to the cytoplasmic (**A**) and membrane (**B**) RCAS1 expression in cancer cells in tumor border parts. Patients with regional (N+) and distant (M+) metastases were included into the analysis.

The probability of survival free from metastasis to regional lymph nodes was higher in patients where RCAS1 expression was absent in the tumor BPs (Figure 5).

### 2.2. The Characteristics of TAMs and CAFs in the Microenvironment: RCAS1 Expression and Cell Number

**Figure 6:**
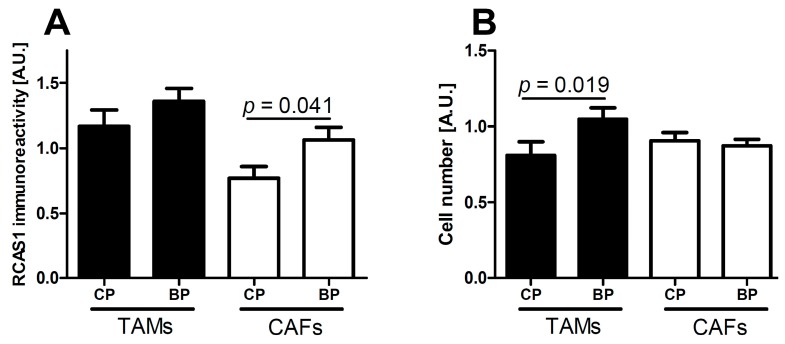
RCAS1 immunoreactivity (**A**) of cells in the tumor microenvironment and number of cells in the tumor microenvironment (**B**) in the central part (CP) and border part (BP) regions of tumors. TAMs: tumor-associated macrophages; CAFs: cancer-associated fibroblasts.

The intensity of RCAS1 expression within fibroblasts was significantly higher in tumor BPs than in tumor CPs (Figure 6A).

The number of tumor-associated macrophages (TAMs) was significantly higher in tumor BPs than in tumor CPs (Figure 6B).

#### 2.2.1. RCAS1 Immunoreactivity and Cell Number Relative to Tumor Grade

**Figure 7:**
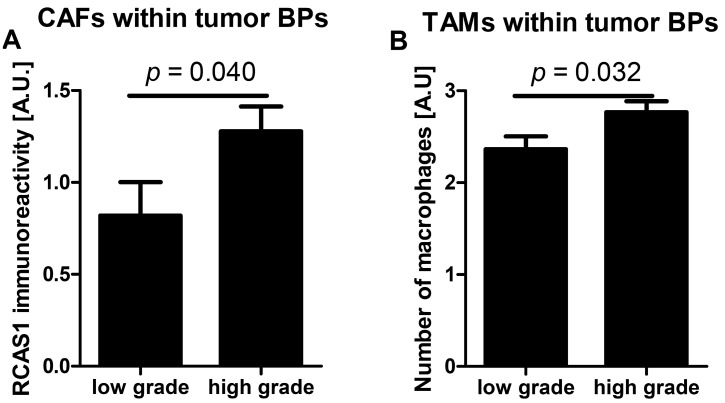
RCAS1 expression in cancer-associated fibroblasts (**A**) and tumor-associated macrophage number (**B**) in the stroma of the border parts (BPs) tumors in relation to tumor grade.

The RCAS1 expression intensity in cancer-associated fibroblasts (CAFs) was significantly higher in the BPs of high-grade tumors compared with low-grade tumors. A significant correlation between high RCAS1 intensity in CAFs and a high grade of the tumor was observed (Figure 7A). The number of macrophages was higher in the BPs of high-grade tumors than in low-grade tumors. A significant correlation was also observed between the number of tumor associated macrophages (TAMs) within the BPs and high grade of the tumor (Figure 7B).

#### 2.2.2. Cell Number Relative to Tumor Stage

**Figure 8:**
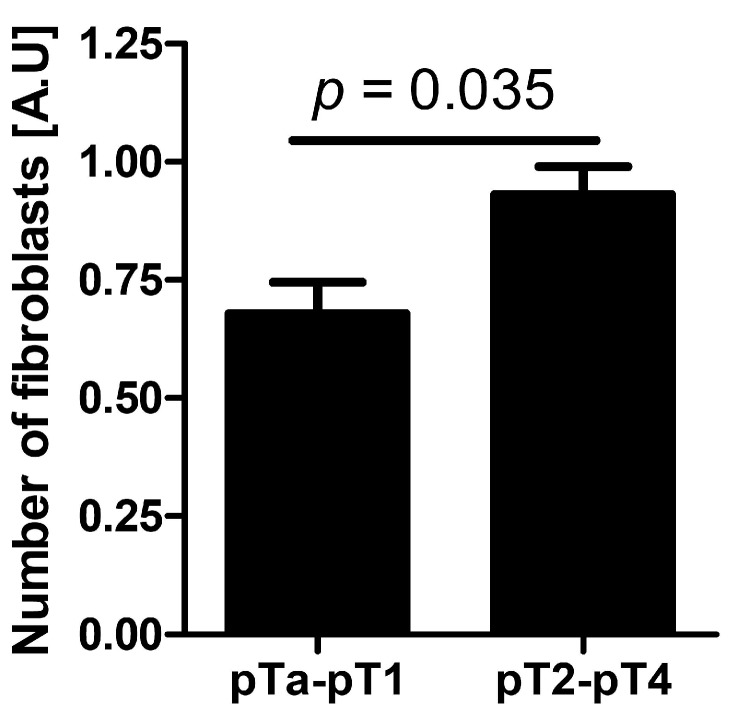
The number of cancer-associated fibroblasts in non-muscle-invasive bladder cancer (stages pTa, pTis, and pT1) and muscle-invasive bladder cancer (stages pT2–pT4).

The number of fibroblasts in the tumor BPs increased with advanced stage of the neoplastic process (pT) and was significant higher for pT2–pT4 tumors (Figure 8).

#### 2.2.3. RCAS1 Immunoreactivity and Cell Number Relative to TIT

**Figure 9:**
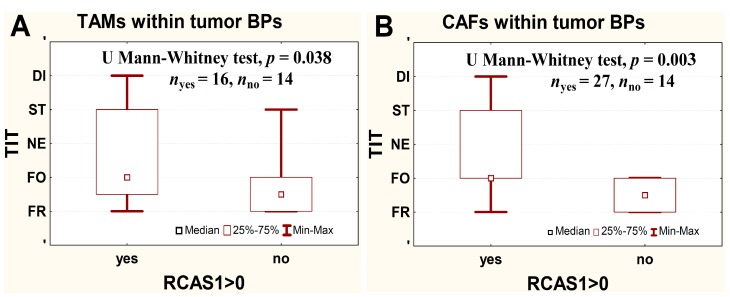
Relationship between RCAS1 expression in border part (BP) tumor-associated macrophages (TAMs) (**A**) and BP cancer-associated fibroblasts (CAFs) (**B**) and the tissue invasion type (TIT) aggressiveness. FR-frontal; FO-focal; NE-nested; ST-styloid; DI-dispersed. Data were analyzed using Mann-Whitney-Wilcoxon test.

The RCAS1 immunoreactivity increased in BP TAMs and in BP CAFs with increasing aggressiveness of TIT (*i.e.*, frontal, focal, nested, styloid, and dispersed) and in tumors without RCAS1 in BP TAMs and in BP CAFs aggressiveness of TIT was significantly lower than in cases with RCAS1 expression (*p* = 0.039 and *p* = 0.004, respectively, Figure 9).

#### 2.2.4. RCAS1 Immunoreactivity Relative to NDN

**Figure 10:**
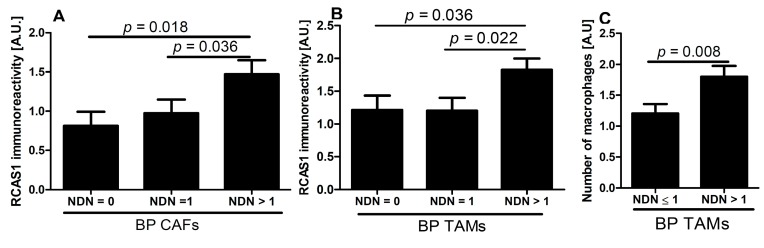
RCAS1 expression in border part (BP) cancer-associated fibroblasts (CAFs) (**A**) and BP tumor-associated macrophages (TAMs) (**B**) increased with increasing NDN. Number of BP TAMs (**C**) was significantly related to the NDN.

Significantly greater immunoexpression of RCAS1 within BP CAFs and BP TAMs was observed in cases with higher NDN (Figure 10A,B), and statistically significant, positive correlation was observed between RCAS1 level and increasing NDN (Spearman rank correlation, *r* = 0.35, *p* = 0.003, and *r* = 0.28, *p* = 0.038, respectively. Similarly, number of BP TAMs increased with increasing number of NDN (Spearman rank correlation, *r* = 0.32, *p* = 0.016) and statistically significant differences were observed between tumors with NDN = 0 or NDN = 1 and cases with NDN > 1 (Figure 10C).

RCAS1 expression in cancer cells was not associated with histological tumor grade, TIT and NDN. RCAS1 in CAFs and TAMs not related to tumor stage metastasis and CAFs and TAMs number within the tumor microenvironment were not related to metastasis.

## 3. Discussion

RCAS1 expression is related to the development of a suppressive cell profile [32] and enables cancer cells to escape the immune system [23]. However, RCAS1 expression is not homogeneous throughout the tumor [19]. In our studies, the BP regions were not identical topographically to the areas that adhered directly to neighboring tissues, but were usually scattered throughout the tumor. We found that the histological differentiation of tumor growth dynamics into the CP and BP areas corresponded topographically to the areas of lower and higher RCAS1 expression, respectively, in both tumor cancer cells (Figure 1) and fibroblasts in the tumor microenvironment (Figure 6A). The formation of the fields with a more dynamic growth, within the tumor areas of an increased RCAS1 expression, suggests the practical implementation of the concept that suppressive microenvironment profile facilitated by RCAS1 molecules, attenuates the immune system inhibition of tumor growth. High expression of the components of the histological pattern of tumor malignancy (G, TIT, NDN, pT, and pN) was observed only in association with RCAS1 expression in tumor BPs (Table 1). This means that all these components are histological indicators of escape from immune surveillance—a higher degree of escape measured by RCAS1 expression is accompanied by greater tumor malignancy related to higher G, TIT, NDN, pT or pN status. The histological tumor malignancy, as indicated by pT and pN, and clinical, as indicated by the metastasis-free survival time, correlated significantly with RCAS1 expression in cancer cells (Figure 3, Figure 4 and Figure 5). Also, malignancy, as indicated by G, TIT, and NDN, correlated with RCAS1 expression in fibroblasts and macrophages in the tumor environment (Figure 8, Figure 9 and Figure 10). These findings suggest that the histological effect of increased RCAS1 expression depends on its cellular source and that RCAS1 expression itself is a component of various signaling pathways. In this study, no component of histological malignancy pattern correlated significantly with RCAS1 expression at the same time in both cancer and stromal cells. These data also suggest that mechanism of tumor escape from immune surveillance depends on RCAS1 expression in both neoplastic cells and cells of tumor environment.

The lack of significant correlations between the grade of tumor histological maturity and RCAS1 expression in cells of squamous cell carcinoma [29,30,33,34], hepatocellular carcinoma [35,36], and intrahepatic bile duct carcinoma [37] has been observed before. Our findings that the RCAS1 expression in cancer cells was not significantly associated with the histological grade of urothelial bladder cancer but that its expression in CAFs in tumor BPs was related to histological grade (Figure 8A) are consistent with those of previous reports. The earlier reports showing a significant negative correlation between the cancer histological maturity grade and RCAS1 expression in tumor cells in gastric [38] and large intestine [39] adenocarcinomas, together with our results on urothelial bladder cancer, may indicate that an organ-specific pathomechanism underlies this relationship.

One of the first locations described for RCAS1-positive macrophages was bone marrow, in which macrophages play an important role in the control erythropoiesis, which involves RCAS1 secretion [40]. Macrophages are involved in the neoplastic process from its earliest stages [41]. By expressing growth factors and proangiogenic factors [42], these cells modify the tumor microenvironment and its progression [43]. Macrophages also release matrix metalloproteinases, which leads to the remodeling of the cancer microenvironment and facilitates cancer cell invasion and migration [44]. In this study, the presence of RCAS1-positive macrophages correlated positively with the infiltrative aggressiveness of urothelial bladder cancer (Figure 9A). This finding has confirmed that the more aggressive TIT forms are related to the cancer-promoting profile of the microenvironment, which involves the participation of macrophages [45].

In the neoplastic process, TAMs usually cooperate with fibroblasts in the cancer microenvironment by releasing transforming growth factor β (TGFβ). At low concentrations, TGFβ acts as a chemoattractant that attracts fibroblasts to the tumor site and, at higher concentrations, it induces fibroblasts to transdifferentiate into myofibroblasts, which are classified as CAFs [46,47]. We cannot exclude the possibility that the greater number of TAMs in BP regions of tumors reflects a more influential role of TAMs in the control of the tumor environment compared with CAFs (Figure 7B). CAFs release factors such as serine proteases, matrix metalloproteinases, and urokinase activator of plasminogen, which degrade the epithelial basement membrane and extracellular matrix, and thereby participate in deciding whether, and with what dynamics, cancer cells can infiltrate the surrounding tissues [48]. In this study, the TIT of greater aggressiveness has correlated with the presence of RCAS1-positive CAFs (Figure 9B), which is consistent with their role in the tumor progression [48,49].

The data available in the literature indicate that TAMs and CAFs participate in the production of metallothioneins [50]. In our study, the presence of nonclassic differentiations correlated significantly with the presence of RCAS1-positive TAMs and CAFs in the tumor microenvironment (Figure 10A,B). This correlation was particularly noticeable for RCAS1-positive TAMs (Figure 10B) and the number of macrophages (Figure 10C) in NDN > 1 tumors. More advanced urothelial bladder cancer showed significant cytoplasmic RCAS1 expression in cancer cells of the BPs in pTa-pT1 and pT2–pT4 tumors (Figure 3A,C–F) and membrane expression in the BPs of pT2–pT4 (Figure 3B,E,F).

There is a lack of agreement in the literature whether RCAS1 expression in tumor cells and tumor advancement are related. Some authors have observed that an increased RCAS1 expression correlates with tumor progression [38,51], while others have found no evidence of such a relationship [43]. We cannot exclude the possibility that the lack of relationship could reflect the inclusion of both BPs and CPs of tumors. In this study, the association between pT and RCAS1 expression in cancer cells was particularly strong in the BPs of urothelial cells infiltrating the bladder muscles (Figure 3B) and in pT2–pT4 tumors a significant increase in the number of CAFs was observed when compared to pTa–pT1 tumors. This suggests that RCAS1 participates in the dynamic production of a microenvironment that favors tumor growth. In urothelial bladder cancer, metastases to lymph nodes correlated significantly with the cytoplasmic (Figure 4A) and membrane (Figure 4B) expression of RCAS1 in cancer cells of the BPs in tumors. We also observed that the probability of lymph nodes metastasis free survival was significantly higher for tumors that did not express RCAS1 in the cancer cells (Figure 5).

## 4. Experimental Section

RCAS1 immunoreactivity was evaluated in samples obtained from 88 patients with urinary bladder cancer (14 women, 74 men). The mean age was 63 years (range 47–84 years) in patients with a noninvasive tumor and 65 years (range 47–80 years) in patients with an invasive tumor. The patients were treated at the Łukaszczyk Oncology Center, Bydgoszcz, Poland, from 2007 to 2010. Tumors were diagnosed and classified according to the International Union Against Cancer TNM Classification criteria [12,52,53]. The grading of these tumors was assessed according to the WHO Classification of Tumours [12]. Patients with N0-Nx tumors were qualified to radical cystectomy and patients with metastases to regional lymph nodes (N+) without clinically detectable metastases (M0) and comorbid serious internal diseases were qualified to radical cystectomy followed by adjuvant chemotherapy, according to Guidelines on Muscle-invasive and Metastatic Bladder Cancer of European Association of Urology [54]. The clinical–pathomorphological characteristics of tumors included in this study are presented in Table 2. Tissue sections were stained using anti-RCAS1 antibody according to the protocol we have described previously [19]. The study was approved by the Committee of Ethics of Scientific Research of Collegium Medicum of Nicolaus Copernicus University, Poland.

**Table 2:** The clinical–pathomorphological characteristic of urothelial bladder tumors.

Feature	Number of Patients
pT	
pTa	3
pTis	1
pT1	16
pT2	19
pT3	32
pT4	17
pN	
pN0	64
pN1–3	24
Grade	
low grade	20
high grade	67
Recurrence	
no	79
yes	9
Second tumor	
absent	61
present	27
CP component	
absent (only BP present)	24
present	64
Concomitant *in situ* tumor	
absent	76
present	12
NDN	
NDN 0	35
NDN 1	21
NDN 2	21
NDN ≥ 3	11
CP TAMs	
absent	25
present	39 *
BP TAMs	
absent	18
present	70
CP CAFs	
absent	33
present	31
BP CAFs	
absent	35
present	53

* In 24 cases, only the BP compartment was present.

### 4.1. Assessment of RCAS1 Expression

Briefly, formalin-fixed paraffin-embedded 4 µm sections were dewaxed and rehydrated, and then incubated with primary anti-RCAS1 antibody (dilution 1:1000; Medical & Biological Laboratories Co., Ltd., Nagoya, Japan) at 4 °C overnight. The sections were incubated for 30 min with EnVision peroxidase-labeled polymer conjugated to anti-mouse secondary antibody (Dako, Carpinteria, CA, USA), and the reaction product was developed for 5 min using 3,3'-diaminobenzidine (DAB) (Dako). After counterstaining with hematoxylin, sections were dehydrated and mounted in a permanent medium (Consul Mount; Thermo Fisher Scientific Inc., Waltham, MA, USA). In tumor cells, RCAS1 expression was evaluated in the cytoplasm and membrane. In the cells in the tumor microenvironment, RCAS1 expression was evaluated in the cytoplasm of CAFs and TAMs.

RCAS1 immunostaining was evaluated semiquantitatively when RCAS1 was seen in ≥50% of cells. The mean staining intensity was evaluated independently by two researchers using the following scale: negative (0) or positive with a grade of 1+ (pale brown), 2+ (brown), or 3+ (dark brown) for positive staining, as described previously [19]. Extreme values for expression intensity were analyzed statistically as 0 (no expression) and 2 and/or 3 (moderate and/or high intensity) for cases that were assessed as 100% by both researchers. To increase the objectivity of the data, an expression intensity of 1, as an indicator of low-intensity RCAS1 expression (most subjective), was rejected and was not included in the statistical analysis. Consecutive sections were stained with hematoxylin and eosin (HE).

### 4.2. Definitions

#### 4.2.1. Mitotic Index and CPs and BPs of Tumors

Within tumor sections, the CPs and BPs were localized precisely based on the mitotic rates, as described previously with slight modifications [30]. Briefly, the BPs were defined as the parts of the tumor with signs of dynamic growth (e.g., presence of small nests of tumor cells, expression of stromal reactivity, inflammatory infiltration, and/or types of stromal modeling pattern). The CPs were defined as the parts of the tumor without signs of dynamic growth.

The mitotic index was defined as the number of cells in mitosis per 1000 neoplastic cells. During measurement of this index, cancer foci with at least twice as much mitotic activity compared with other foci within one tumor were identified as tumor BPs. The mean mitotic index within CPs was assessed as 2.2 for pTa-pT1 and 2.6 for deep-infiltrating tumors (pT2–pT4). The BP of the tumors was defined as an area with a higher mitotic index, and the mean mitotic indexes were 3.8 and 5.1 for pTa–pT1 and pT2–pT4, respectively. For the latter, differences between CPs and BPs were significant (*p* = 0.0001; Figure 2).

#### 4.2.2. NDN of Tumors

The NDN is a feature of tumor biology that reflects the tendency toward divergent histological differentiation. The tendency of tumor histological composition was estimated by the presence of nonclassic differentiation, which was estimated as described previously [12].

#### 4.2.3. TIT of Tumors

**Figure 11:**
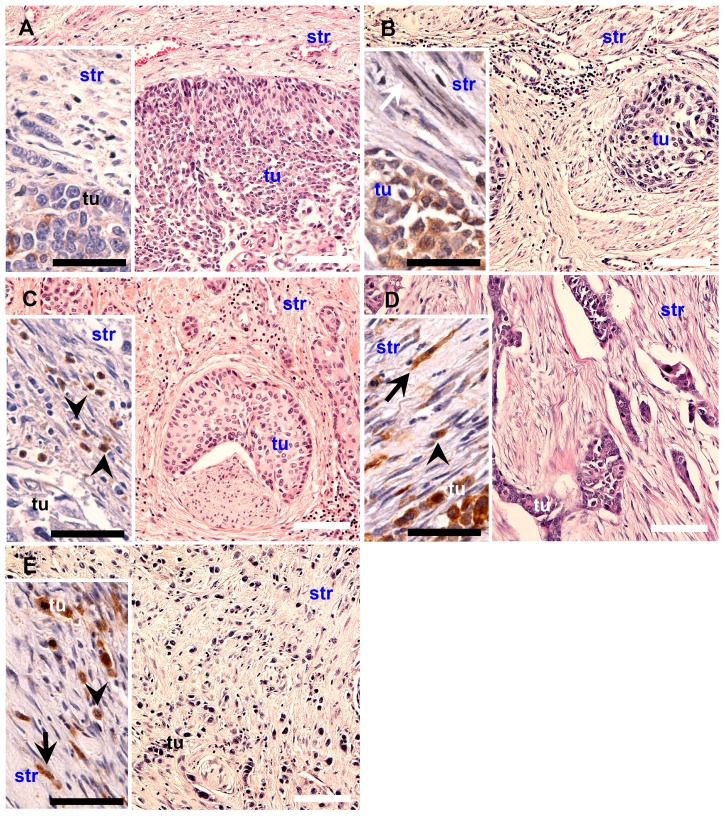
Types of invasion in sequence reflecting the increasing histological malignancy of the tumor: **A**-FR, **B**-FO, **C**-NE, **D**-ST, and **E**-DI type. Inserts represents RCAS1 immunostaining in stromal cells: arrows indicate fibroblasts, arrow heads–macrophages. White scale bars: 100 µm, black scale bars: 50 µm.

Cancer cell infiltration as a component of local tumor spread was in H&E stained sections. The types of tissue invasion were classified as described previously with slight modifications [18]. Briefly, frontal was defined as the type of invasion with the distinct border between the tumor and its nearest tissue vicinity in a more or less regular line. Focal was defined as an invasion with a linear border but with a segmented penetrating tumor environment that appeared as groups of cohesive tumor cells. Nested was defined as a border lacking linear structure in which a group of cohesive tumor cells penetrated the tumor environment and did not have a connection with the main mass of tumor, but formed nests Styloid was defined as microenvironment penetration through spaces of small histological resistance, with characteristic digital formations of cohesive tumor cells but without signs of histological maturation. Dispersed was defined as microenvironment penetration by noncohesive small groups of tumors or single tumor cells (Figure 11). The TIT was evaluated using a classification scale ranging from the least to the most aggressive type of invasion; *i.e.*, frontal, focal, nested, styloid, and dispersed.

### 4.3. Identification of TAMs and CAFs and Assessment of Their Numbers

TAMs and CAFs were identified using immunohistochemistry: anti-CD68 (clone PG-M1) for TAMs and anti-α-smooth muscle actin (αSMA) for CAFs [55,56]. These cells were identified using the manufacturer’s protocols using PT-Link for antigen retrieval and the slide-processing instrument Autostainer Link for immunostaining (Dako). CAFs were counted per each high-power field (HPF) using the following scale: 0, no or a few cells; 1, tens of cells; 2, a few hundred cells. TAMs were counted per each HPF using the following scale: 0, no cells; 1, one cell; 2, several cells; and 3, tens of cells.

### 4.4. Statistical Analysis

The relationships between RCAS1 and variables such as tumor grade, pT, TIT, NDN, and the ability to metastasize were analyzed using Mann-Whitney-Wilcoxon test. The regional (N+) and distant metastasis (M+) free survival time was assessed with Kaplan–Meier curves. The statistical analyses were performed using STATISTICA data analysis software (version 8.0; StatSoft, Inc., Tulsa, OK, USA) and Prism (version 4.00; GraphPad Software, San Diego, CA, USA). A *p* value <0.05 was considered to be significant.

## 5. Conclusions

Heterogeneity of RCAS1 immunoreactivity within a tumor and its microenvironment cells is related to the tumor’s histological structure. The histological pattern of tumor malignancy, as described by its grade, TIT, NDN, pT, and pN, is promoted by the immune escape mechanism, and RCAS1 appears to be involved in this process. Escape occurs in the tumor’s BPs, where RCAS1 expression increases in tumor cancer cells and in the stroma cells—TAMs and CAFs. This results in acceleration of tumor growth and, consequently, in a progression of its histological phenotype to a more malignant form. Therefore, the histological pattern of tumor malignancy appears to be a morphological reflection of the escape from immune regulation-more advanced escape, more malignant behavior. The best measure of a capability to escape immune regulations is probably the RCAS1 expression-dependent pattern of tumor growth, which is related to the development of a immunosuppressive microenvironment. This mechanism implies that the lack of immunological control facilitates the development of a new tumor structure and attendant changes in the histological pattern (G, NDN, TIT, pT). These changes are neither the result nor the source of tumor malignancy but rather a reflection of its histology. Tumor malignancy may not necessary has its reflection in the histological pattern, being in agreement with the observation that the clinical course is not always in line with the histological presentation. The histological manifestation may represent a freeze-frame containing information about the progress of tumor escape from immune regulations. Our results suggest that mechanism of tumor escape from immune surveillance depends on RCAS1 expression in both neoplastic cells and cells of tumor environment. This type of analysis will enable individualized prediction and prognosis of tumors by taking into account the local status of the immune system. Further studies are needed to confirm these results in a larger number of cases and to obtain more detailed knowledge about the role of signal-transduction pathways and RCAS1 involvement in determining the histological pattern of urothelial bladder cancer malignancy.
